# Effect of Wei Qi Booster on immune and anti-oxidative function in aged mice

**DOI:** 10.3389/fvets.2024.1446770

**Published:** 2024-07-24

**Authors:** Shuang Ma, Yuming Chen, Zhilong Zhou, Aituan Ma

**Affiliations:** ^1^College of Life Sciences and Food Engineering, Hebei University of Engineering, Handan, Heibei, China; ^2^College of Traditional Chinese Veterinary Medicine, Hebei Agricultural University, Baoding, Hebei, China

**Keywords:** Traditional Chinese Veterinary Medicine, Wei Qi Booster, aged mice, immune, anti-oxidative

## Abstract

This research was conducted to examine the impact of Wei Qi Booster (WQB) on immune parameters and anti-oxidative function in aged mice. Fifty aged mice were randomly assigned to five different groups. Group A was designated as the control group. Mice in Group B were receiving Levamisole at 10 mg/kg body weight. Each mouse in groups C, D and E received 0.1, 1, and 2% WQB, respectively. Another ten young mice, designated as group F, were fed regularly. The mice were fed according to the above methods for 28 days. Results showed that relative to the control group, the body weight and immune organs indexes experienced a substantial rise in the group with 1% WQB. In addition, 1% WQB could improve the activity of SOD and reduce the MDA levels. Expressions of CD4 and sIgA increased while CD8 decreased in the jejunum of aged mice treated with WQB. IL2 and IFN-γ levels increased in the 1% WQB group, showing no notable difference compared to the young mice group. The results demonstrated that WQB can elevate immune levels and enhance anti-oxidative functions in aged mice.

## 1 Introduction

The immune system comprises immune organs, cells and molecules. Its roles consist of recognizing and eliminating antigenic invaders and collaborating with other bodily systems to uphold the equilibrium and physiological harmony of the internal milieu. Immunodeficiency pertains to the incapacity of the body’s immune system to execute its usual tasks in safeguarding the host. And immunodeficiencies are significantly more prevalent, as they are associated with other systemic conditions such as diabetes, malnutrition, aging and oxidative stress. Therefore, it is crucial to discover medication that can minimize aging and oxidative stress. The consumption of natural products rich in antioxidant phytochemicals is becoming increasingly popular, with these products being seen as having fewer side effects. Wei Qi Booster (WQB) is a modification of the classical formula Si Jun Zi Tang. WQB consist of Dang Shen (*Codonopsis pilosula*), Huang Qi (Astragalus membranaceus), Wu Yao (Lindera aggregata), Chen Pi (*Citrus reticulata*), Dang Gui (Angelica sinensis), Xuan Shen (Scrophularia ningpoensis), Bai Hua She She Cao (Hedyotis diffusa) and Ban Zhi Lian (Scutellaria barbata), which can be used to tonify Wei Qi, Qi and Blood, and clear Heat-Toxins. Many researchers have reported that WQB can reduce upper respiratory infections and enhance IFN-γ and IL-2 levels in chickens (1, 2). Many pharmacological studies have showed that the herbs in WQB could enhance body immunity and exert anti-tumor effects. One paper reported that Dang Shen restored the splenic index and normalized the concentrations of IFN-γ and IL-2. Additionally, it increased ileum secretory immunoglobulin A (sIgA) in cecum (3). Liu and his colleges demonstrated that *Codonopsis pilosula* polysaccharide possesses cancer-fighting, antiviral, anti-inflammatory, immune-regulating, antioxidant, and various other biological properties. It also decreases the MDA in liver homogenate and increases the level of SOD (4). Research has showed that Huang Qi has a range of biological functions, including cancer-fighting, immunomodulatory, anti-oxidant, anti-diabetes, anti-microbial, and inflammation-reducing activities (5, 6). Liu reported that Huang Qi may also shield immune organs, leading to a noticeable rise in the spleen index, substantial increases in the thymus index, and elevated proportions of CD4^+^ cells (7). The Chinese herbal Wu Yao has been commonly utilized in ancient Chinese herbal therapy to address a range of health issues, such as inflammatory, liver injury. It has also been observed to enhance the histopathological condition while reducing serum concentrations of ALT, AST, TG, TC, and MDA in the liver. These protective effects are associated with its anti-oxidative action (8). Cao (9) have shown that Dang Gui notably reduced Bax expression while boosting Bcl-2 levels. Additionally, it increased SOD levels and decreased MDA levels.

There are few reports on the modification of the classical formula Si Jun Zi Tang and its effects on immune and anti-oxidative activities. This research provides novel insights into developing more rational and efficient herbal remedies for immunity enhancement and antioxidation based on TCVM.

## 2 Materials and methods

### 2.1 Animals and housing conditions

Sixty Kunming mice (of Clean Grade) were obtained from the Hebei Medical University (Shijiazhuang, China). Prior to the experiment, all mice were given one week to acclimatize in environmentally regulated enclosures where the temperature was adjusted to 23°C, and a consistent light and dark cycle lasting 12 h each was upheld. During this period, food and water were given without limitation. The experiment was performed following the ethical principles suggested by the Council for Animal Care in Hebei province.

### 2.2 Handling of animal

**Figure 1:**
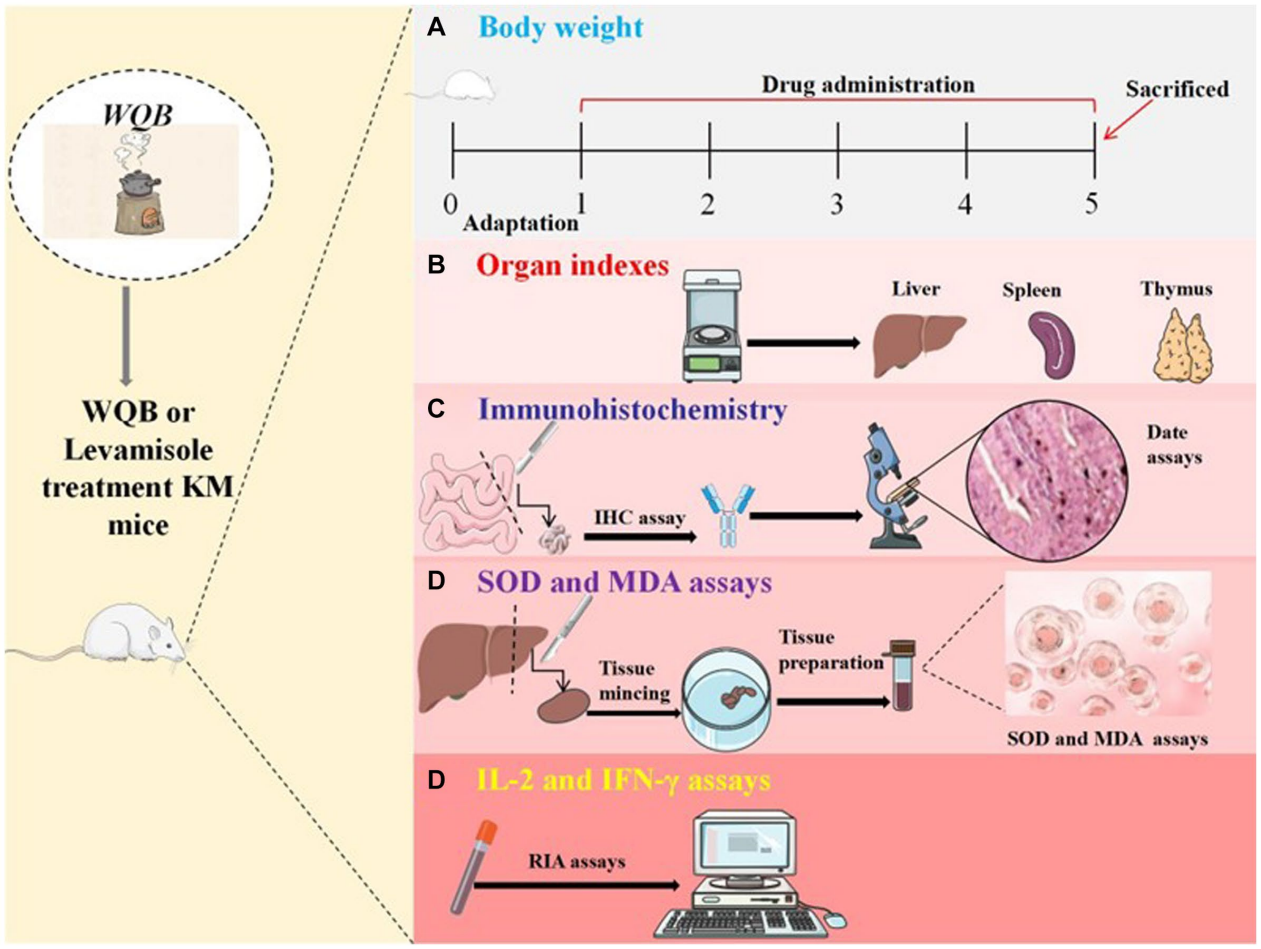
Experimental procedure. Kunming mice were orally administered either WQB or Levamisole for four weeks. Mice in control group were orally fed as well. The body weights were recorded throughout the trial **(A)**. After four weeks of administration, organ indexes were measured **(B)**, and the expressions of CD4, CD8, and sIgA in cells of duodenum and jejunum were analyzed using IHC **(C)**. The concentrations of MDA and SOD in the liver were measured **(D)**, and the content of IL-2 and IFN-γ in the serum were analyzed using RIA **(E)**.

Fifty aged mice were evenly distributed into five categories. Group A functioned as the control group. Mice in Group B received Levamisole at a dosage of 10 mg/kg body weight (BW) (Jinpai Pharmaceutical, China). Each mouse in groups C, D and E received WQB (Dr. Xie’s Jing Tang Herbal Inc., Reddick, FL USA) mixed in feed at concentrations of 0.1, 1 and 2%, respectively. Another ten young mice were fed a standard diet and designated as group F. The mice were fed according to the aforementioned methods for a duration of 28 days. Twenty-four hours after the final treatment, following four weeks of treatment, all the mice underwent anesthesia and were subsequently euthanized. The intact liver, spleen and thymus tissues were delicately dissociated and extracted. Subsequently, they were rinsed with PBS, and the organ indexes (organ weight/body weight) were calculated. Liver tissue was homogenized using a buffer solution. Moreover, the duodenum and jejunum were meticulously dissected and excised, rinsed with PBS, and subsequently fixed in Bouin’s solution for immunohistochemistry (IHC) examination. Hematological samples were acquired by puncturing the orbital vein. Following centrifugation at a speed of 3,000 × g for a duration of 20 min at 4°C, the serum was successfully separated. The serum was subsequently preserved at −80°C until evaluations (Figure 1).

### 2.3 Performance in growth

For each group, live body weight (LBW) was monitored over a span of 4 weeks. The body weight gain (BWG) for each group was calculated during the trial.

### 2.4 IHC assays

After being fixed in Bouin’s solution and subsequently embedded in paraffin, the jejunum specimens were sectioned into tissue slices with a thickness of 5 μm. For IHC staining, the jejunum samples were subjected to an overnight incubation at a temperature of 4°C, utilizing primary antibody of CD4 or CD8 that was diluted at a ratio of 1:500. The duodenum specimens were left to incubate at 4°C overnight with a 1:100 ratio of the primary sIgA antibody. The CD4 and CD8 antibodies were purchased from Biolegend (State of California, USA). The sIgA antibody was purchased from ZSGB-BIO (Beijing, China). The tissue slices were subsequently incubated at ambient temperature with the goat anti-rabbit IgG conjugated with HRP. Half an hour following the application of the detection system, the reaction was visualized using DAB combined with hydrogen peroxide. The specimens were scrutinized by the Olympus IX71 Research Inverted Phase microscope (Olympus Co., Tokyo, Japan), and the concentration was measured with Image J software (National Institute of Health, USA).

### 2.5 MDA and SOD assays

The hepatic samples, weighing 200 mg, were homogenized, and then subjected to centrifugation for 5 min at 1000 g in an ice-cold environment to prepare a 10% homogenized liver tissue solution. The homogenized mixture was centrifuged for a quarter-hour at 1000 g and 4°C to obtain the supernatant for later biochemical analysis. MDA and SOD concentrations were assessed according to the specifications of the kit (Jiancheng Bioengineering, Nanjing, China).

### 2.6 Assay for IL-2 and IFN-γ

Serum (100 μL) was measured for IL-2 and IFN-γ using the RIA kit (Jiancheng Bioengineering, Nanjing, China).

### 2.7 Statistical analysis

The collected data from the experiment were subjected to statistical analysis using SPSS 26.0 software. Group differences were assessed using two-sided unpaired Student’s *t*-test. A *p*-value less than 0.05 was deemed to indicate statistical significance.

## 3 Results

### 3.1 Body weight

**Figure 2:**
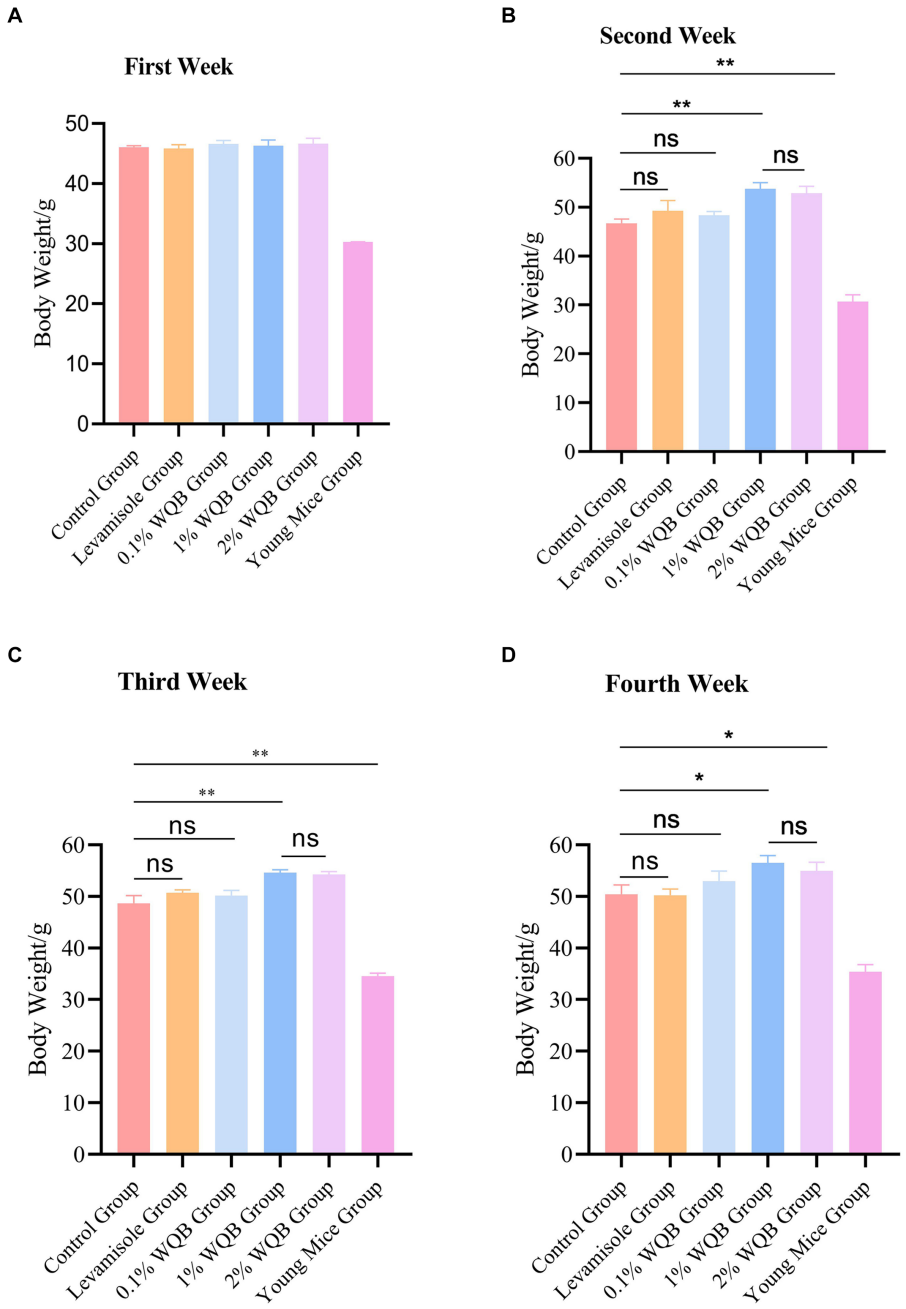
The body weight of mice in each group. **(A)** First week. **(B)** Second week. **(C)** Third week. **(D)** Fourth week. ns, not significant; *p < 0.05; **p < 0.01.

After 28 days of intervention, the weights of body in each group were measured (Figure 2). There is no difference of body weight between the control group and other groups (Figure 2A). Statistically, the body weight in the control group were markedly lower than those observed in the 1 and 2% WQB group (*p* < 0.01, *p* < 0.05) during the second (Figure 2B) and third week (Figure 2C), as well as the fourth week (Figure 2D). Additionally, there were no substantial variations in body weight among the 1% WQB group and 2% WQB group (*p* > 0.05) (Figures 2B–D). Consequently, WQB (1, 2%) may increase the body weight of aged mice (Figure 2).

### 3.2 Organ indexes

**Figure 3:**
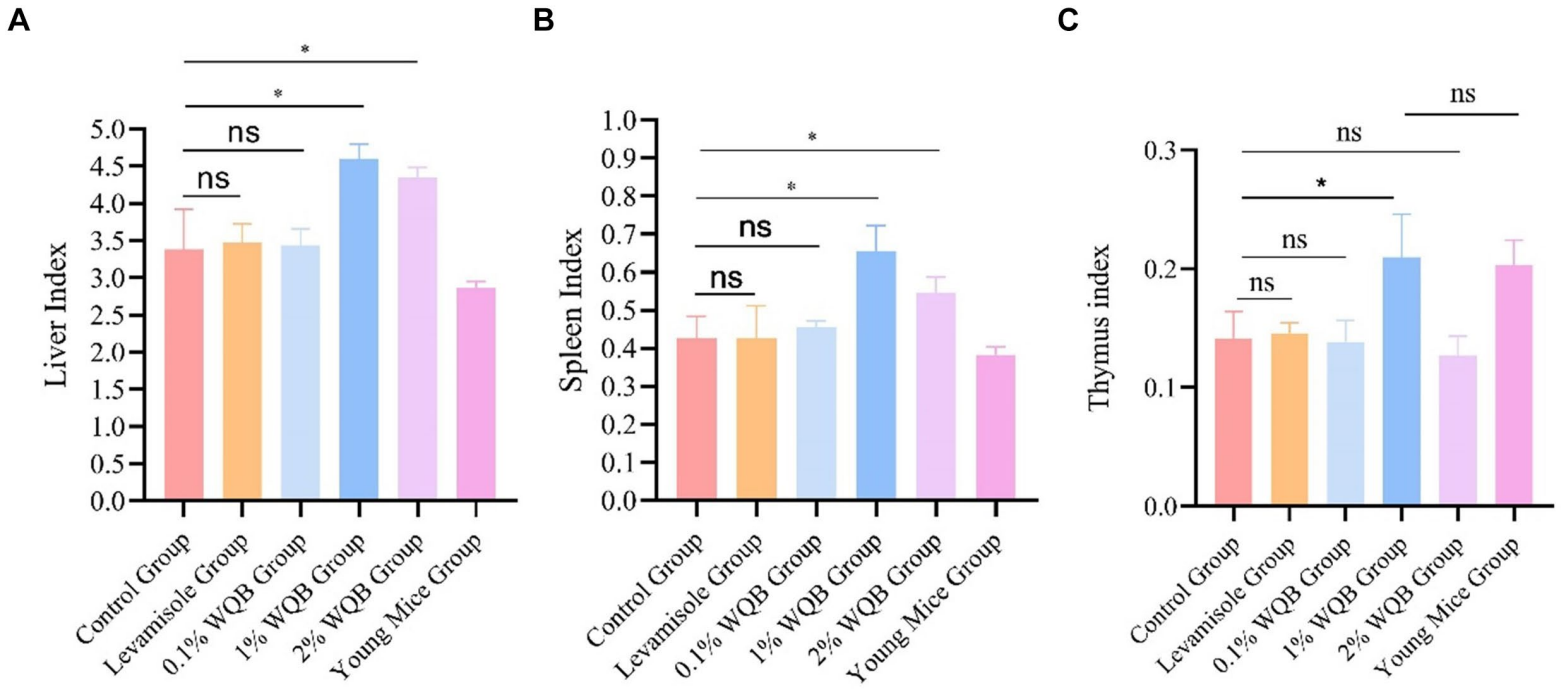
Analysis of organ indexes of mice. **(A)** Liver index. **(B)** Spleen index. **(C)** Thymus index. ns, not significant; *p < 0.05.

The fundamental immune activity was demonstrated by the indices of the liver, spleen, and thymus. A significant rise in liver index (Figure 3A) and spleen index (Figure 3B) was observed in the mice of the 1 and 2% WQB groups in comparison with the control set (*p* < 0.05). Figure 3C shows that the thymus index in mice from the 1% WQB group exceeded that of the mice in the control group (*p* < 0.05).

### 3.3 IHC results

**Figure 4:**
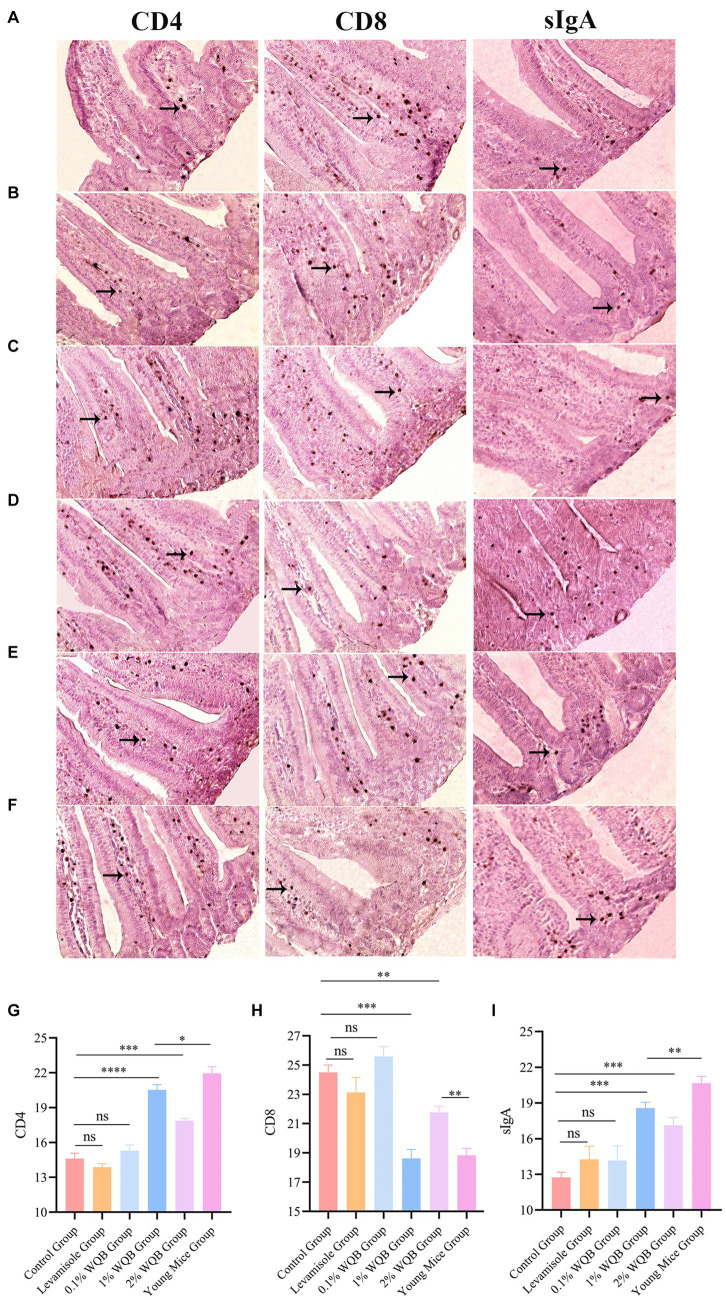
IHC analysis of mice intestinal mucosa. Cells expressing CD4, CD8 and sIgA proteins were indicated by arrows. **(A)** Control group. **(B)** Levamisole group. **(C)** 0.1% WQB group. **(D)** 1% WQB group. **(E)** 2% WQB group. **(F)** Young mice group. **(G)** Expression levels of CD4. **(H)** Expression levels of CD8. (I) Expression levels of sIgA. ns, not significant; *p < 0.05; **p < 0.01; ***p < 0.001; ****p < 0.0001.

To further examine the impact of WQB on CD4, CD8 and sIgA expressions in mice, IHC was performed. The results revealed protein expressions of CD4, CD8 and sIgA in the lamina propria of intestinal mucosa (Figures 4A–F). No significant alterations were observed in the concentrations of CD4, CD8, and sIgA between the Levamisole group, the 0.1% WQB group, and the control group (Figures 4G–I). The quantities of CD4 and sIgA exhibited notable increases in both the 1 and 2% WQB groups in contrast to the control group (*p* < 0.01, *p* < 0.001) (Figures 4G–I).

### 3.4 MDA and SOD

**Figure 5:**
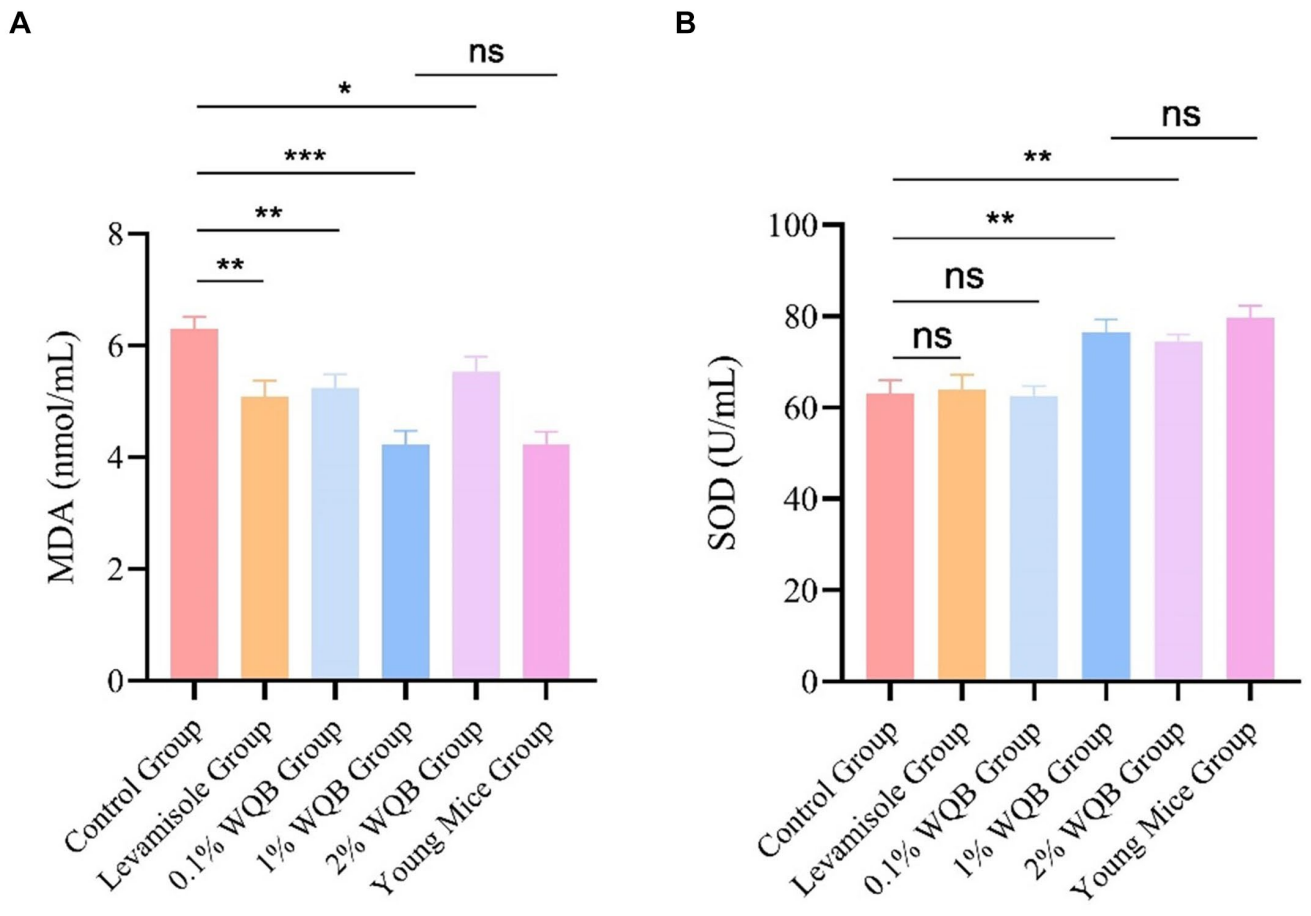
Analysis of MDA and SOD in liver. **(A)** The level of MDA. **(B)** The level of SOD. ns, not significant; *p < 0.05; **p < 0.01; ***p < 0.001.

Notably, in comparison to mice in the control group, the addition of WQB resulted in a reduction in the concentration of MDA (Figure 5A), with the 1% WQB group exhibiting the lowest levels compared to the control group (*p* < 0.001) (Figure 5A). SOD exhibited lower expression levels in the control group as opposed to the 1 and 2% WQB groups (*p* < 0.01) (Figure 5B).

### 3.5 IL-2 and IFN-γ

**Figure 6:**
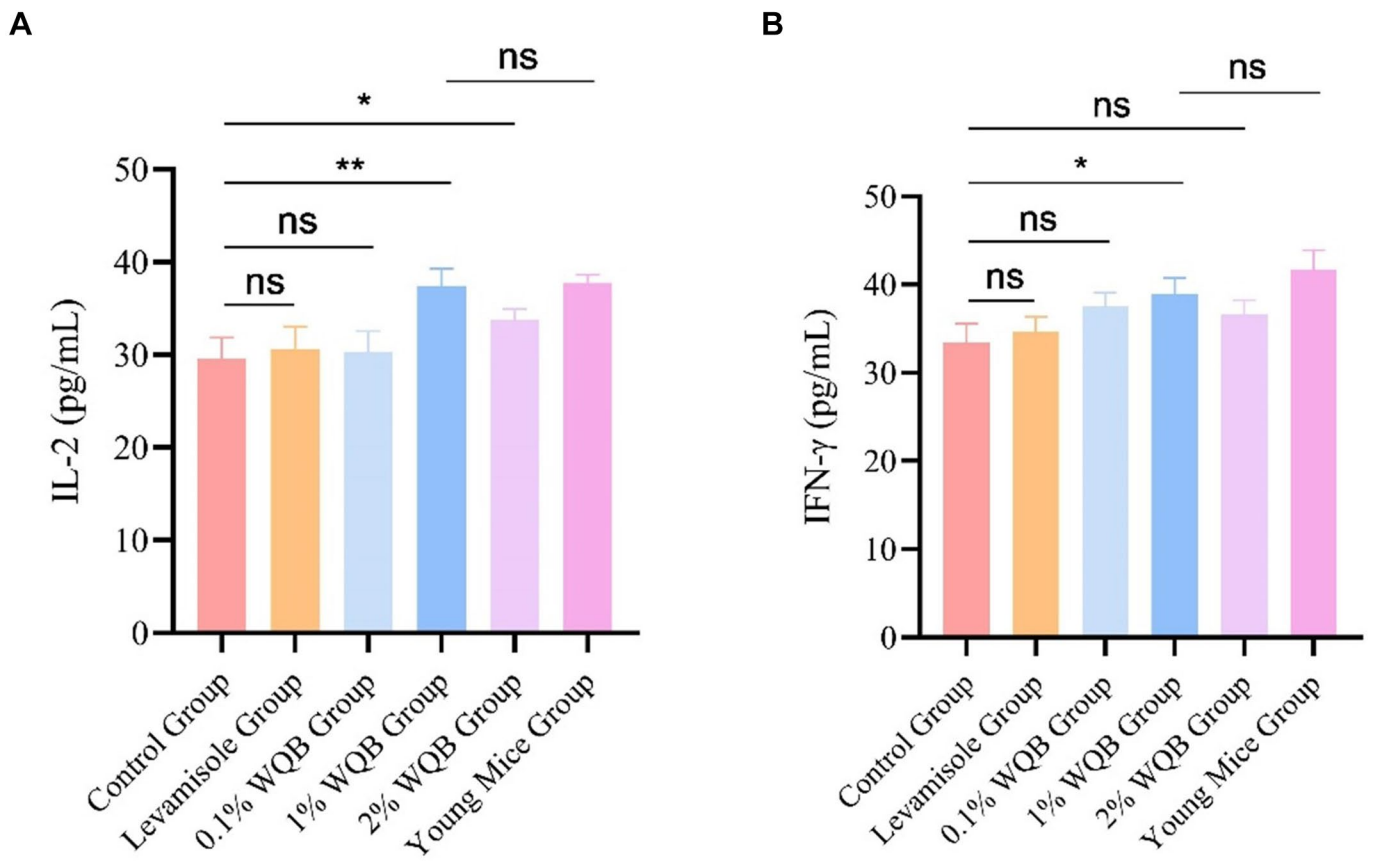
Analysis of IL-2 and IFN-γ in serum. **(A)** The concentration of IL-2. **(B)** The concentration of IFN-γ. ns, not significant; *p < 0.05; **p < 0.01.

Treatment with 1 and 2% WQB increased the expression of IL-2 (*p* < 0.05, *p* < 0.01). However, there was no significant contrast between the group of young mice group and the group administered with 1% WQB (*p* > 0.05) (Figure 6A). Meanwhile, mice subjected to 1% WQB displayed heightened levels of IFN-γ in contrast to those in the control cohort (Figure 6B).

## 4 Discussion

The World Health Organization has framed the well-being and medical services for senior individuals based on the notion of aging well. Healthy aging is characterized as “the progression of fostering and sustaining the ability to perform effectively in later stages of life, facilitating overall wellness” (10). As people grow older, the physiological robustness of bodily organs gradually wanes, leading to organ vulnerability and age-associated issues like diminished skeletal muscle mass, cardiac enlargement, reduced cardiac performance, and osteoporosis (11, 12). The process of getting older is impacted by a multitude of intricate elements, and there is not a lone objective or solitary medication that can adequately decelerate the advancement of aging and ailments associated with aging. The aim of this investigation was to assess the effect of WQB on immune markers and antioxidant function in elderly mice. Our findings revealed that WQB administration ameliorates oxidative stress and immune dysfunction in elderly mice.

The intestine carries out numerous physiological functions, such as nutrient absorption, secretion, excretion, and immune regulation. Hence, it is crucial to study the aging intestine. The advancement of TCM offers a novel approach for treating patients with immune system issues and oxidative damage. SJZT is a traditional Chinese medicine formulation known for its anti-aging properties and ability to treat age-related diseases. For instance, it has been shown to alleviate aging in the spleen and spleen deficiency syndrome (13, 14). Researchers discovered that Si Jun Zi Tang diminishes respiratory tract inflammation in mice experiencing chronic asthma by regulating the mTORC1 pathway (15). Liang et al. initially investigated the mechanism of Si Jun Zi Tang in combating aging utilizing network pharmacology (16). As a frequently prescribed treatment in clinical practice, SJZT has demonstrated its efficacy in postponing the progression of aging and enhancing their quality of life. Nonetheless, the mechanism by which WQB affects aging patients remains unclear. Therefore, we conducted experiments to validate the potential mechanism of WQB in enhancing anti-aging development and immune system function.

WQB is a modified version of the traditional herbal formula Si Jun Zi Tang, which consists of eight medical herbs. These herbs include Huang Qi (Tonifies Qi in whole body and Wei Qi), Dang Gui (Tonifies Blood), Dang Shen (Tonifies Qi and boosts Wei Qi), Wu Yao (Moves Qi and clears Stagnation), and so on (17). Multiple studies have demonstrated that Huang Qi exhibits potential in boosting antioxidant functions, reducing inflammation stress, and influencing autophagy responses in humans and other mammals (18). Moreover, Huang Qi has been shown to protect the epithelial barrier by actively engaging immune cells, including macrophages and dendritic cells (19). Contemporary pharmacological investigations have additionally indicated that Dang Gui is not only harmless and non-toxic, but it also possesses anticancer, antioxidant, immune-modulating, and neuroprotective characteristics. Studies indicate that incorporating diets supplemented with astragalus-derived polysaccharides can increase the variety of cecal microorganisms in broiler (20). Dang Gui can aid in preserving the cohesion of the gut lining and also demonstrate a beneficial effect on gut immune function. Feeding broilers with 3% Dang Gui supplements enhanced development efficiency and gut health, bolstering the mechanical, chemical, and immune protections of the intestine (21). Dang Shen is a widely recognized herbaceous perennial native to Northern China, celebrated for its health-promoting qualities and similarities to ginseng. The polysaccharide constituents have exhibited a range of biological functions, including anti-cancer and immune-regulating effects (22–24). Wu Yao, an evergreen shrub prevalent across China, has extracts that have been documented to exhibit antioxidant, antidiabetic, and anticancer properties (25). Furthermore, as a medicinal herb, Wu Yao has been noted for its efficacy in treating gastrointestinal, metabolic, inflammatory, and urinary system disorders (26). Flavonoids from Wu Yao, administered at doses ranging from 50 to 200 mg/kg, demonstrated significant reductions in ALT and AST activities, along with decreased MDA concentrations and increased SOD activity, leading to an overall enhancement in antioxidant levels in the serum of mice with CCl_4_-induced acute liver damage (27). Experimental results indicate that WQB can enhance the indices of various organs. Furthermore, using IHC and ELISA assays, we demonstrated that WQB can lower CD8 and MDA levels while restoring CD4, sIgA, SOD, IL-2, and IFN-γ expression. Consequently, WQB presents a promising therapeutic approach for enhancing immune function and mitigating oxidative damage in mice, warranting further investigation and potential clinical application.

As people age, their immune system typically suffers significant impairment and deterioration, a process known as immunosenescence. Furthermore, impaired immune function is a key indicator of aging. Of all the immune organs, the thymus, a primary organ of the lymphoid system, is essential for producing naive T cells, which are critical for both cellular and humoral defense (28). The gradual decline of the thymus with age is regarded as a major factor contributing to the decline in immune capability (29). The spleen typically participates in controlling humoral immunity (30). The process of bodily aging is closely linked to the functions of both the thymus and spleen. Thus, evaluating the body’s immune function by examining the thymus and spleen is a critical indicator. Moreover, the most prominent alterations in immune aging include a substantial decrease in naive T cells and a buildup of memory T cells, a lowered ratio of CD4/CD8 T cells, a diminished quantity of B cells, and a rise in circulating inflammatory signaling molecules. The emergence of inflammation alongside aging is a multifaceted developmental phenomenon. Typically, inflammation escalates with age, irrespective of acute infection or additional physiological strain (31). Research indicates that aging, oxidative stress (32), and inflammation are interconnected and mutually influential (33). Aging typically involves reduced immunity and alterations in both the intestines and immune system, making the quest to slow aging and enhance the quality of life for the elderly a valuable area of research. The thymus and spleen serve as primary centers for immune activity and act as natural defenses against invasions. The thymus and spleen index are useful indicators of the body’s immune status to some degree (34). Investigations have revealed that aging can limit the proliferation of immune cells and hinder the development of immune organs, thereby weakening the body’s capacity to generate an effective immune response (35). Nevertheless, pharmaceutical intervention can positively influence both the thymus and spleen index. The research findings show that the thymus and spleen indices in the aged model group are considerably reduced compared to the 1% WQB group. This suggests that the defensive organs in the elderly model exhibit notable atrophy, reflecting a decline in their immune function. Following a 4-week period of WQB treatment, the thymus and spleen ratio of the elderly model showed a notable increase compared to those of the Aged group, suggesting that WQB exerted a beneficial influence on the defensive structures of elderly rodents. Furthermore, the amalgamation of Rb1 and Re yielded superior outcomes compared to single administrations. Moreover, through the assessment of the Liver index and the rate of body weight gain, it was observed that the administration of 1% WQB significantly boosted the body weight gain rate and Liver index. This suggests that the physiological capabilities of the aging model were successfully rejuvenated. IgA serves as a crucial element within antibodies, with its concentration indicating the robustness of the body’s immune response (36). Additionally, the impacts of WQB on the IgA levels in the jejunum were examined. The findings indicated a notable elevation in IgA levels in aging models following the administration of WQB. Upon encountering an organism, our immune system promptly generates IgA, serving as the initial line of defense or delaying infection. The discoveries of this investigation unveiled a notable rise in IgA levels following 4 weeks of WQB administration, suggesting that WQB could provide enduring protection against infections, thus indicating long-term enhancement of immunity. The thymus affects T cell generation and migration to the periphery, thereby affecting T cell pool diversity (37). IL-2 modulates the function of white blood cells accountable for immune responses. IL-2 exerts its role by attaching to the IL-2 receptor found on lymphocytes (38). The main sources of IL-2 are activated CD4^+^ and CD8^+^ T lymphocytes. IL-2 serves as a significant immune booster, stimulating the expansion of particular T cell subsets and thereby enhancing overall immune function in the body (39). In elderly mouse models, IL-2, CD4 and INF-γ were markedly diminished, whereas CD8 were substantially increased. Treatment with 1% WQB significantly restored IL-2, CD4 and INF-γ levels, demonstrating WQB’s potential to boost immune function in aged mice. The enzyme SOD is a vital part of the enzymatic antioxidant network, critical for decreasing and averting oxidative stress through its protective antioxidant functions. Antioxidant enzymes help neutralize harmful electrophiles, enabling cells to maintain their internal environment to some extent even at lower mercury levels. SOD eliminates superoxide radicals. This research demonstrated that WQB lowered MDA levels, an oxidative stress marker, while increasing SOD levels, an antioxidant, in mouse liver tissue. Our findings suggest that WQB can mitigate oxidative damage in mice.

## 5 Conclusion

WQB is a modification of the traditional Chinese formula Si Jun Zi Tang by adding several Chinese herbals so as to additionally strengthen its immunoregulatory function. The findings of this study demonstrated that WQB could increase CD4 and sIgA but decrease CD8 expressions in jejunum of aged mice. Levels of IL-2 and INF-γ in the blood of aged mice were mildly upregulated by WQB. Besides, MDA level was downregulated, and SOD was upregulated in the liver, showing the antioxidant activity of WQB. In conclusion, WQB exerts both immunoregulatory and antioxidative functions in aged mice.

## Data availability statement

The raw data supporting the conclusions of this article will be made available by the authors, without undue reservation.

## Ethics statement

The animal study was approved by the Council for Animal Care in Hebei province. The study was conducted in accordance with the local legislation and institutional requirements.

## Author contributions

SM: Conceptualization, Data curation, Investigation, Methodology, Writing – original draft, Writing – review & editing. YC: Investigation, Methodology, Software, Writing – review & editing. ZZ: Data curation, Investigation, Writing – original draft. AM: Conceptualization, Supervision, Writing – review & editing.
